# Coupling Aptamers to Short Interfering RNAs as Therapeutics

**DOI:** 10.3390/ph4111434

**Published:** 2011-10-27

**Authors:** Laura Cerchia, Carla Lucia Esposito, Simona Camorani, Silvia Catuogno, Vittorio de Franciscis

**Affiliations:** 1 Istituto per l'Endocrinologia e l'Oncologia Sperimentale del CNR “G. Salvatore”, Via S. Pansini 5, 80131 Naples, Italy; 2 Dipartimento di Biologia e Patologia Cellulare e Molecolare, Università di Napoli “Federico II”, Via S. Pansini 5, 80131 Naples, Italy

**Keywords:** aptamer, intracellular delivery, microRNA, small interfering RNA

## Abstract

RNA-based approaches are among the most promising strategies aimed at developing safer and more effective therapeutics. RNA therapeutics include small non-coding miRNAs, small interfering RNA, RNA aptamers and more recently, small activating RNAs. However, major barriers exist to the use of RNAs as therapeutics such as resistance to nucleases present in biological fluids, poor chemical stability, need of specific cell targeted delivery and easy entry into the cell. Such issues have been addressed by several recent reports that show the possibility of introducing chemical modifications in small RNAs to stabilize the molecular conformation and increase by several fold their integrity, while still preserving the functional activity. Further, several aptamers have been developed as excellent candidates for the specific recognition of cell surface targets. In the last few years, by taking advantage of recent advances in the small RNA field, molecular bioconjugates have been designed that permit specific targeting and may act as cargoes for cell internalization of small RNAs acting on gene expression that will be discussed in this review.

## 1. Introduction

Innovative targeted therapeutic strategies aim at developing new molecules with high target affinity and specificity with suitable pharmacokinetic properties for *in vivo* applications. From this optic short non-coding RNAs were revealed to be attractive molecules. In the last decades significant advances have been attained in the knowledge of molecular mechanisms leading to selective inhibition of gene expression and protein function. However, in order to successfully translate RNA-based therapeutics to the clinic several challenges must be addressed, including appropriate stability in biological fluids, high efficiency and specificity of delivery, durable safety and target selectivity.

Several classes of molecules have been characterized with potential applications as RNA therapeutics in the treatment of human diseases. These include ribozymes, RNA decoys, aptamers, small interfering RNA (siRNA) and microRNA (miRNA) [1].

The discovery of RNA-mediated interference (RNAi) for gene silencing has provided a powerful tool for loss-of-function studies and therapeutic opportunities [2,3]. RNA interference is a natural process of gene specific silencing that occurs in organisms ranging from plants to mammals as a defense against viruses. si/miRNAs are formed from longer precursor molecules as short double-stranded RNAs (dsRNAs) of 20–24 base pairs [4]. One strand that directs silencing is the *guide* strand while the other strand, named the *passenger*, is degraded. In the cytoplasm, the RNA-induced silencing complex (RISC) drives the *guide* strand of the dsRNA to hybridize with the target mRNA to prevent translation or induce degradation depending on the degree of complementarity [5]. Base pairing between siRNAs and their targets generally shows full complementarity whereas, with the exception of the 2–8 bases *seed* region at the 5′ terminus, miRNAs usually show partial complementarity with their targets. miRNAs have the capacity to target multiple genes simultaneously and regulate important biological processes including, transcription, cell cycle, cell growth, proliferation and apoptosis. They have been shown to be involved in the pathogenesis of diverse diseases including cancer, stroke, diabetes, diseases of the liver, kidney, and cardiovascular system as well as neurodegenerative and infectious diseases [6-8]. On the other hand siRNAs are the best characterized RNA-based reagents that have been developed for several disease including cancer, kidney, ocular, retinal and metabolic disorders.

As a difference with siRNAs and miRNAs, the function of ribozymes and aptamers doesn't involve the formation of the RISC. The hammerhead small ribozymes are nucleolytic oligonucleotides that recognize and excise a given target RNA molecule [9]. Aptamers constitutes an emerging attractive class of therapeutic molecules able to tightly bind to specific protein or non-protein targets by folding into complex tertiary structures [10,11].

Recognition by toll-like receptors (TLRs) in immune cells represents a major obstacle to the use of RNA-based therapeutics. However, immune recognition the immune response of single stranded siRNAs (ss-siRNA) or ds-siRNAs by TLRs can be bypassed by the replacement of only uridines with their 2′-fluoro, 2′-deoxy, or 2′-*O*-methyl modified counterparts without reducing their silencing potency [12-15]. In addition, immunogenicity has been found to be either absent or limited when 1,000-fold higher doses of a nucleic acid aptamer than would be required clinically were administered to monkeys [16]. This property depends on the fact that antibodies to synthetic oligonucleotides are not generally produced and, in addition, the innate immunity response against non-self RNAs does not hinder aptamer therapy because 2′-modified nucleotides abrogate TLRs responses [17].

A major impediment to the clinical development of RNA drugs is the lack of an appropriate and high efficiency *in vivo* delivery strategy to guarantee intracellular target accessibility and specificity of delivery. The use of viral vectors, despite their high efficiency, has been impaired greatly due to the associated mutagenicity or oncogenesis, several host immune responses, and high cost of production. Therefore, non-viral vectors continue to draw significant attention despite their low efficacy.

## 2. RNA-Based Therapies

Currently, the list of oligonucleotides of therapeutic interest is growing rapidly with over one hundred clinical trials and two therapeutic oligonucleotides that have been already approved by U.S. Food and Drug Administration (FDA) and marketed, the Vitravene antisense antiviral and the Macugen RNA-based aptamer. Two classes of therapeutic oligonucleotides have predominantly been developed: siRNA and aptamers, and several of them are currently in clinical trials (Table 1).

### 2.1. siRNAs

Recently, the use of RNAi-based gene silencing has been demonstrated in humans for treatment of several diseases, as discussed in multiple recent reviews [18]. We report here only few not exhaustive examples of the possible therapeutic applications.

Various clinical studies have explored the direct tissue delivery of siRNA into the eye for macular degeneration in humans. Among the growth factors implicated in the age-related macular degeneration (AMD) process, the vascular endothelial growth factor (VEGF) has been shown to be a major inducer of choroidal neovascularization [19]. Several studies have recently addressed the silencing of VEGF [20,21] or the VEGF receptor 1 (VEGFR1) [22] by RNA interference (RNAi) using either intravitreous/periocular injection of siRNA or using adenovirus backbones to allow stable endogenous transgene expression of short hairpin (sh)RNAs resulting in a potent reduction of VEGF or VEGFR1.

Silencing of gene expression by RNAi has been extensively studied to develop innovative cancer therapeutic strategies. Indeed, many of the siRNAs are in different stages of development for the treatment of different kind of tumors. For examples, among the siRNA therapeutics for the treatment of solid tumors, CALAA-01 and Atu027, targeting the M2 subunit of ribonucleotide reductase and protein kinase N3, respectively, are in Phase I, whereas, FANG against Furin is in Phase II. Further, SPC2996 against BCL-2 is in Phase II for treatment of chronic myeloid leukemia (from http://ClinicalTrials.gov). As shown in Table 1, the number of possible applications of RNAi therapeutics are growing rapidly and now include also viral infections, respiratory, brain, skin and metabolic diseases.

In recent studies, given the strong impact of siRNAs for therapeutic applications, a great effort is focused on the optimization of the efficacy of the siRNAs through relatively minor chemical and structural modifications to canonical siRNA. The final aim is to improve loading of the guide strand into the RNAi machinery and reduce off-target effects and competition with endogenous miRNAs.

The group of Rossi [23-26] has reported pioneering studies demonstrating that Dicer substrate interfering RNA (dsiRNA) are more potent than classical synthetic 21-mer siRNAs, showing more robust formation of a high molecular weight complex known to contain Dicer and TRBP (two primary members of the RISC-loading complex).

### 2.2. Aptamers

Aptamers are short single-stranded DNAs or RNAs that like antibodies, bind with high affinity to specific targets by folding into complex tertiary structures. They have some important advantages over antibodies and other protein-based reagents as therapeutics. A number of these advantages stem from the fact that aptamers are generated by an iterative *in vitro* evolution procedure named Systematic Evolution of Ligands by EXponential enrichment (SELEX) avoiding the use of animals or cells. In addition, aptamers can be readily chemically modified to enhance their bioavailability and pharmacokinetics [27-29]. Further, as discussed above, another important advantage of RNA aptamers over proteins is the fact that RNA is much less immunogenic than proteins [16].

The list of aptamers against important therapeutic targets is growing rapidly and some of them have already entered the clinical pipeline (see Table 1) for the treatment of different diseases [30-32]. The most successful therapeutic application of an aptamer is represented by Macugen (or pegaptanib, marketed by Eyetech Pharmaceuticals/Pfizer), an RNA-aptamer that binds and antagonizes the action of VEGF. The aptamer has been fully approved by the FDA in December 2004 for the treatment of exudative AMD. In order to translate this aptamer into the clinic, it has been chemically modified with 2′-fluoropyrimidines (2′-F-Py), 2′-*O*-Me-purines (2′-*O*-Me-Pu) and polyethylene glycol (PEG) to generate a better therapeutic agent [33,34].

Many other aptamers, not yet approved by the FDA, are currently in clinical trials. For example other two aptamers, named E10030 and ARC1905, are in Phase II and I of clinical trials for the treatment of AMD, respectively. E10030 is a DNA-aptamer directed against the platelet-derived growth factor-B (PDGF-B) chemical modified with 2′-F-Py and 2′-OMe-Pu and PEG [35]; while ARC1905 is a RNA-aptamer targeting the complement component 5 (C5) containing 2′-F-Py and PEG [36,37].

Furthermore, different aptamers targeting blood-clotting factors seems to be effective anticoagulant agents. The ARC1779 is a DNA-aptamer directed against the A1 domain of von Willebrand factor, currently in phase II clinical trials for the treatment of thrombotic microangiopathies (TMA) [38,39]; while Nu172 is a chemical unmodified DNA-aptamer directed against thrombin, currently in phase II clinical trials to evaluate its potential use as an anticoagulant during acute coronary artery bypass surgery.

Particularly interesting is REG-1, an aptamer targeting the coagulation factor IXa. This is the first case of a modulator-controlled aptamer able to provide a time-controllable therapy. REG-1 is a two-part therapeutic agent, consisting of an RNA aptamer specific for the coagulation factor IXa (RB006) and a single stranded RNA oligonucleotide complementary to the RB006 aptamer (RB007). Aptamer inhibition of the factor IXa by RB006 is structurally disrupted by administration of the antidote complementary strand RB007. The REG-1 aptamer-antidote therapy has been tested in Phase I and II clinical trials with promising results as an anticoagulation therapy to prevent clot formation during cardiac surgery [40].

Moreover, different aptamers for cancer therapy are also in clinical trials. NOX-A12 is an L-RNA spiegelmer directed against the stromal cell-derived factor-1α (SDF-1α), a chemokine which attracts and activates immune and non-immune cells that bind to chemokine receptors CXCR4 and CXCR7. This aptamer is in Phase I clinical trials for the treatment of hematologic tumors. The AS1411 aptamer, instead, showed effectiveness for the treatment of acute myeloid leukaemia (AML) in phase I and II clinical trials. AS1411 is a DNA-aptamer, directed against nucleolin [41], a protein often overexpressed on the surface of cancer cells. This DNA aptamer is part of the guanine-rich oligonucleotide class of aptamers that form G-quartets, a structural element that exhibits antiproliferative activity. Nucleolin has many functions, so inhibiting this protein with AS1411 affects a variety of signaling pathways, including NF-κB [42] and Bcl-2 [43].

Apart from the aptamers mentioned above, many other aptamers are not yet developed in clinic but target molecules of high therapeutic interest thus appearing as excellent drug candidates for a wide range of human pathologies [30].

### 2.3. miRNAs

Although the clinical development of miRNAs has not yet been realized, they are attractive candidates as prognostic biomarkers and therapeutic targets in different diseases including cardiovascular disease and cancer. In addition, the use of complementary antisense oligonucleotides has been developed for miRNA silencing in research and therapy. Antisense inhibitors act by competing for miRNA binding to the proper sites on target mRNAs and include small synthetic RNAs, antagomir, and modified RNA oligonucleotides, as locked nucleic acid (LNA) [44].

Cardiovascular disease is the leading cause of death in industrialized nations. Several miRNAs have been recently implicated in cardiomyocyte hypertrophy, increased fibrosis and apoptosis during heart failure [45]. Using mice with induced cardiac hypertrophy it has been recently shown that miR21 is upregulated in hearth fibroblasts, increases the extracellular signal-related kinases (ERKs)-mitogen-activated protein kinase (MAPK) activity and regulates cell survival and growth factor secretion. Cardiac hypertrophy and fibrosis can be attenuated and even prevented by the administration of a specific antagomir that suppresses miR-21 levels and reduces cardiac ERK-MAPK activity [46]. On the other hand, has been shown that miR-199a expression is sensitive to low oxygen levels and is rapidly downregulated in cardiac myocytes to undetectable levels, thus rapidly resulting in increased levels of mRNA target, hypoxia-inducible factor (Hif)-1alpha. Conversely, restoring miR-199a levels during hypoxia inhibits Hif-1alpha expression, reduces apoptosis and protects the cells from hypoxic injury [47]. Cardiac remodeling can be as well prevented by the administration of an inhibitory antagomir for the cardiac-specific miR-208a thus improving the overall survival of treated rats [48]. All together, these studies indicate the potential of RNA-based therapies for cardiovascular diseases.

Expression of several miRNAs has been shown to be deregulated in many cancer types. Further, based on their involvement in basic cellular functions, miRNAs may act as oncogenes (oncomirs) or tumor suppressor as critical players in cell transformation [49-51].

For example, it has been demonstrated that the let-7 family contains miRNAs regulating the RAS family of oncogenes [52]. Petrocca *et al.* [53] showed that the miR-106b-25 cluster plays a key role in gastric cancer interfering with proteins involved both in cell cycle and apoptosis. In other studies, miR-155 was found overexpressed in Hodgkin lymphoma, pediatric Burkitt lymphoma and diffuse large B-cell Lymphoma [54-56]; miR-143 and miR-145 were significantly downregulated in colon cancer tissue compared with colonic mucosa [57]; miR-21 was overexpressed in many tumors [49], including glioblastoma [58], cholangiocarcinoma [59], multiple myeloma cells [60] and breast cancer [61,62]. Moreover, studies that investigated the expression of the entire microRNAome in various human solid tumors and hematologic malignancies have revealed differences in miRNA expression profiling between neoplastic and normal tissues [63-66]. miRNAs play a key role also in tumor metastasis. Indeed, for example miR-139 suppresses metastasis of hepatocellular carcinoma, while miR10-b was found highly expressed in metastatic breast cancer cells [67,68] even if its clinical utility is still questioned [69].

## 3. RNA-Based Bioconjugates Molecules for siRNA Delivery

Potent sequence selective gene inhibition by siRNA ‘targeted’ therapeutics promises the ultimate level of specificity, but siRNA therapeutics is hindered by poor intracellular uptake, thus efficient delivery strategies remains the main challenge for their clinical development [70].

In this respect a promising application of aptamers is to use them to deliver a variety of secondary reagents, including therapeutic siRNAs, specifically to a targeted cell population (Table 2) [71,72].

This means that aptamers function as specific recognition ligands to target cells, which is especially significant given the whole cell-SELEX strategy to target specifically cell surface epitopes [73]. Once delivered, the secondary reagents would then impart their therapeutic effect to this subset of cells within the treated individual. Because non-targeted cells would not be exposed to the secondary reagent, the potential for unwanted side-effects such as death of normal cells is substantially reduced.

The cell-SELEX method allows for the generation of aptamers against cell surface targets by replicating the native conformation and glycosylation pattern of the extracellular regions of proteins. Recently, multiple groups have reported selections using living cells as the target to identify receptor-specific aptamers and those that bind to a specific cell type [73,74]. Some of these aptamers have been used as delivery cargos to target cells giving the cell-type specific expression of cell surface proteins on cell populations of therapeutic value.

**Figure 1.**
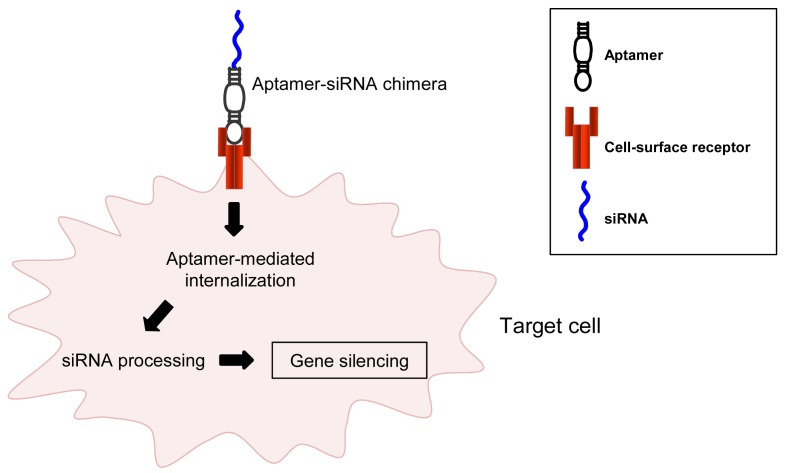
Aptamers as delivery agents. Aptamers that bind to cell surface receptors can be used to deliver siRNA to target cells.

In the aptamer-based delivery approach, the last goal is to develop an aptamer to the extracellular portion of such a protein and then use the aptamer to deliver the secondary reagent to the targeted cell population via binding the targeted protein on the surface of the targeted cell type. Because this binding in some cases also results in the endocytosis of the aptamer/secondary reagent complex, this approach can be used to deliver reagents such as siRNAs that depend on delivery to intracellular compartments for their proper function (Figure 1).

**Figure 2.**
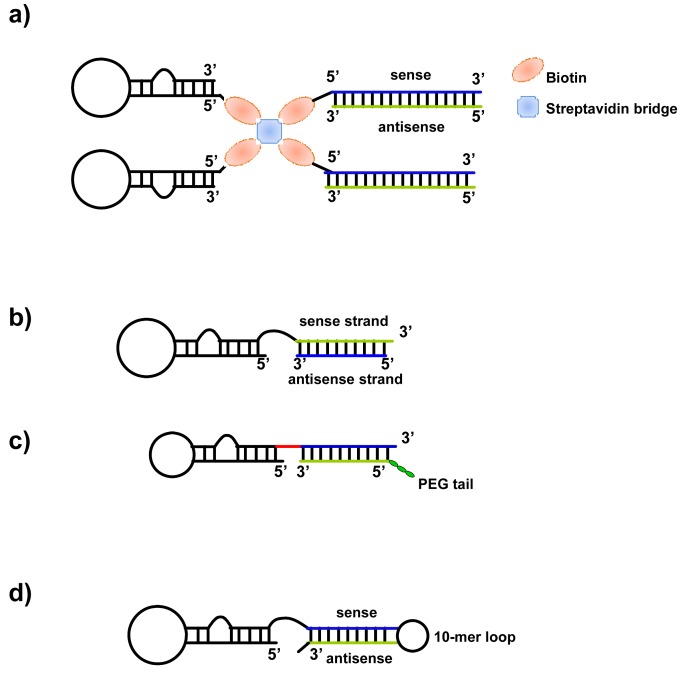
Anti-PSMA aptamer-siRNA chimeras. (**a**) The RNA duplex and RNA aptamers are chemically conjugated with biotin. Thus, two biotinylated siRNAs and two aptamers are non-covalently assembled via streptavidin; (**b**) The 3′ end of the aptamer is extended to contain the nucleotide sequence that is complementary to the antisense strand of the siRNA, and the chimera is formed by annealing the aptamer to the siRNA antisense strand; (**c**) optimized chimeras in which the aptamer portion of the chimera is truncated, and the sense and antisense strands of the siRNA portion are swapped. A two-nucleotide 3′-overhang and a PEG tail are added to the chimera; (**d**) the 3′-terminus of the aptamer is conjugated to the sense strand of the siRNA, followed by a 10-mer loop sequence and then by the antisense strand of the siRNA.

To date, the best-characterized aptamers for targeted delivery are the two 2′-F-Py-RNA aptamers (A9 and A10) that have been generated against the extracellular domain of the prostate-specific membrane antigen (PSMA) [75]. These aptamers bind with high affinity to the acinar epithelial cells of prostate cancer tissue. They have been used to deliver not only siRNA, but also nanoparticles, quantum dots (QDs) and toxins to prostate cancer cells [73]. Different approaches in which PSMA-aptamer has been linked to siRNAs have been reported (Figure 2).

A first study reports the non-covalent conjugation of siRNA with A9 aptamer via a streptavidin connector [76]. The 27mer Dicer substrates targeting laminin A/C and GAPDH genes and the RNA aptamers were chemically conjugated with biotin. Thus, two biotinylated siRNAs and two aptamers were non-covalently assembled via a streptavidin bridge. The resulting conjugates were incubated with PSMA-positive LNCaP cells without any further preparation, and were taken up within 30 min. The inhibition of gene expression was mediated by the aptamers and as efficient as observed with conventional lipid-based reagents.

In the same year, McNamara *et al*. [77] described the generation of the anti-PSMA A10 aptamer-siRNA chimeras. The 3′ end of the aptamer was extended to contain the nucleotidic sequence complementary to the antisense strand of siRNA targeting the polo-like kinase 1 (PLK1) and BCL-2 survival genes, and the chimera was formed by annealing the aptamer to the siRNA antisense strand. The resulting chimeras were effective in silencing target genes and inducing cell death specifically in PSMA-positive cancer cells.

In addition, the PSMA-siRNA chimeric molecule has been further modified for *in vivo* application [78]. The aptamer portion of the chimera was truncated, and the sense and antisense strands of the siRNA portion were swapped. A two-nucleotide 3′-overhang and a PEG tail were added to the chimera. The modified chimera was able to inhibit prostate cancer xenograft growth when administrated systemically.

To date several groups have adapted the covalent assembly approach to aptamer-mediated siRNA delivery [72]. In these studies, the anti-PSMA A10 aptamer has been conjugated to siRNAs against eukaryotic elongation factor (EEF)2 [79] and two key components of the nonsense-mediated mRNA decoy (NMD) [80]. In addition, since short hairpin RNAs (shRNAs), like miRNA precursors are better substrates for Dicer, Ni *et al*. [81] linked a shRNA against the DNA-activated protein kinase (DNA-PK) to a truncated A10 aptamer (A10-3) generating a single intact nuclease-stabilized 2′ fluoro-modified pyrimidine molecule. The 3′-terminus of the A10 aptamer was conjugated to the passenger (sense) strand of the siRNA, followed by a 10-mer loop sequence and then by the guide or silencing (antisense) strand of the siRNA.

Rossi and colleagues have extensively characterized the HIV glycoprotein gp120 as a target for aptamer-mediated siRNA delivery [82-84]. In these studies, an inhibitory RNA aptamer targeting the HIV envelope protein gp 120, has been used to deliver attached anti-HIV *tat/rev* siRNAs into HIV infected cells via binding to envelope expressed on the cell surface, resulting in internalization of the aptamer and delivery of a dicer substrate siRNA to RISC. *In vivo* delivery of the aptamer and aptamer-siRNA conjugates into a humanized mouse model for HIV infection suppressed HIV replication and completely protected T-cells from HIV mediated T-cell killing.

With the development of the conjugation strategies, the list of aptamers against surface epitopes that are being used as delivery agents is growing rapidly and now includes those against PTK7 [85,86], nucleolin [87], mucin 1 [88,89], and EGFR [90] the have been used to deliver not only siRNA, but also nanoparticles, quantum dots (QDs), toxin and chemiotherapeutics to target cells (see Table 2).

## 4. Market and Perspectives

Even if only one nucleic acid aptamer has been approved and is on the market, aptamers hold an extraordinary potential in drug development and it is plausible that the global interest for their development will increase in the next few years. Accordingly, a new technical market research report, from BCC Research [91], estimated that the global aptamer market value of $236 million in 2010 will grow to nearly $1.9 billion in 2014, for a 4-year compound annual growth rate of 67.5%.

To date, Archemix Corp. is a leading biopharmaceutical company in the development of aptamers as therapeutics. It is the owner of the aptamer technology patent and it collaborates with other pharmaceutical companies (Regado, Antisoma, ARCA Biopharma and Ophthotech) to develop and commercialize a pipeline of partnered aptamers in the cardiovascular disease, hematology and oncology areas.

Moreover, the development of aptamers as delivery agents for therapeutic RNAs can have a considerable impact on aptamer market in the near future. Indeed, intracellular delivery has been a key challenge for RNA modalities and the potential of bringing together the properties of aptamers and microRNA therapeutics will allow to overcome this limitation and open further potential for RNA-based therapeutics.

Recently Archemix Corp. started a collaboration with miRagen Therapeutics Inc., a biopharmaceutical company focused on developing innovative microRNA-based therapeutics for cardiovascular and muscle disease, for the development of conjugated aptamer-microRNA molecules capable of intracellular delivery and subsequent microRNA targeting. Combining aptamers and microRNA therapeutics has the potential to solve the intracellular delivery challenge for certain RNA-based therapeutic approaches. In this perspective, even if aptamer-miRNA chimeras have not been already described in literature, it is plausible that the approaches discussed in this review for aptamer-siRNA conjugation could be as well adapted to generate aptamer-miRNA molecules of fundamental therapeutic value.

## Figures and Tables

**Table 1.** siRNAs and aptamers in clinical trials.

	**Name**	**Company**	**Target (s)**	**Therapeutic Indication**	**Clinical Stage**
**siRNAs**	TD101	Pachyonychia Congenita Project	Keratin 6A N171K mutant	Pachyonychia congenita	Phase I
	QPI-1007	Quark Pharmaceuticals	Caspase 2	Non-arteritic anterior ischaemic optic neuropathy	Phase I
	AGN211745	Sirna Therapeutics	VEGFR1	AMD Choroidal neovascularization	Phase II
	PF-655	Quark	RTP801	Diabetic macular oedema (DME), AMD	Phase I
	SYL040012	Sylentis	β2 adrenergic receptor	Glaucoma	Phase II
	CEQ508	MDRNA	β-catenin	Familial adenomatous polyposis	Phase I
	ALN-PLK1	Alnyam Pharmaceuticals	PLK1	Liver tumours	Phase I
	FANG	Gradalis	Furin	Solid tumours	Phase II
	CALAA-01	Calando Pharmaceuticals	RRM2	Solid tumours	Phase I
	SPC2996	Santaris Pharm.	BCL-2	Chronic myeloid leukaemia	Phase II
	ALN-VSP02	Alnylam Pharmaceuticals	VEGF, kinesin spindle protein	Solid tumours	Phase I
	NCT00672542	Duke University	LMP2, LMP7, and MECL1	Metastatic melanoma	Phase I
	Atu027	Silence Therapeutics	PKN3	Advanced, recurrent or metastatic solid malignancies	Phase I
	QPI-1002/I5NP	Quark Pharmaceuticals	p53	Acute kidney injury	Phase II
	TKM-ApoB	Tekmira Pharmaceuticals Corp.	APOB	Hypercholesterolaemia	Phase I
	PRO-040,201	Tekmira Pharmaceuticals Corp.	APOB	Hypercholesterolaemia	Phase I
	SPC3649	Santaris Pharma	miR-122	Hepatitis C virus	Phase II
	pHIV7-shI-TAR-CCR5RZ	City of Hope Medical Center/Benitec	HIV Tat protein, HIV TAR RNA, human CCR5	HIV	Phase 0
	ALN-RSV01	Alnylam Pharmaceuticals	RSV nucleocapsid	RSV in volunteers	Phase II
**Aptamers**	Macugen (Pegaptanib)	Eyetech Pharmaceuticals/Pfitzer	VEGF-165	AMD Diabetc retinopathy	Approved Phase III
	E10030	Ophthotech Corp./Archemix Corp.	PDGF-B	AMD	Phase II
	ARC1905	Ophthotech Corp./Archemix Corp.	C5	AMD	Phase I
	ARC1779	Archemix Corp.	vWF	TMA	Phase II
	NU172	ARCA Biopharma/Archemix Corp.	Thrombin	Acute coronary artery bypass surgery	Phase II
	REG-1 (RB006/RB007)	Regado Biosciences/Archemix Corp.	Factor IXa	Percutaneous coronary intervention	Phase II
	NOX-A12	NOXXON Pharma	SDF-1α	Lymphoma patients (undergoing autologous stem cell transplantation)	Phase I
	NOX-E36	NOXXON Pharma	CCL2	Type 2 diabetes and diabetic Nephropathy	Phase I
	AS1411 (AGRO001)	Antisoma/Archemix Corp.	Nucleolin	AML	Phase II

**Table 2.** Aptamers as delivery tools.

**Aptamer composition**	**Target**	**Cargos/targeted delivery**	**Therapeutic Indication**
RNA, 2′-F-Py	PSMA	siRNA, Toxin, QDs, nanoparticles and chemiotherapeutics	Prostate cancer therapy

RNA, 2′-F-Py	gp120	siRNA	HIV infection

RNA, 2′-F-Py	CD4	siRNA	HIV infection

RNA	EGFR	Au NPs	Cancer

DNA	PTK7	Doxorubicin, Au-Ag NPs	Cancer

DNA	Mucin 1	QDs, photodynamic therapy agents	Cancer

DNA	Nucleolin	QDs	Cancer
